# A Validation Study Comparing Risk Prediction Models of IgA Nephropathy

**DOI:** 10.3389/fimmu.2021.753901

**Published:** 2021-10-15

**Authors:** Yan Ouyang, Zhanzheng Zhao, Guisen Li, Huimin Luo, Feifei Xu, Leping Shao, Zijin Chen, Shuwen Yu, Yuanmeng Jin, Jing Xu, Manman Shi, Hafiz Muhammad Jafar Hussain, Wen Du, Zhengying Fang, Xiaoxia Pan, Weiming Wang, Jingyuan Xie, Nan Chen

**Affiliations:** ^1^ Department of Nephrology, Institute of Nephrology, Ruijin Hospital, Shanghai Jiao Tong University School of Medicine, Shanghai, China; ^2^ Department of Nephrology, The First Affiliated Hospital of Zhengzhou University, Zhengzhou, China; ^3^ Department of Nephrology, Sichuan Provincial People’s Hospital, Chengdu, China; ^4^ Department of Nephrology, The First People’s Hospital of Yunnan Province, Kunming, China; ^5^ Department of Nephrology, The First Affiliated Hospital of Wenzhou Medical University, Wenzhou, China; ^6^ Department of Nephrology, Qingdao Municipal Hospital, Qingdao, China

**Keywords:** IgA nephropathy, disease progression, end-stage renal disease, risk prediction models, risk factor

## Abstract

We aimed to validate three IgAN risk models proposed by an international collaborative study and another CKD risk model generated by an extended CKD cohort with our multicenter Chinese IgAN cohort. Biopsy-proven IgAN patients with an eGFR ≥15 ml/min/1.73 m^2^ at baseline and a minimum follow-up of 6 months were enrolled. The primary outcomes were a composite outcome (50% decline in eGFR or ESRD) and ESRD. The performance of those models was assessed using discrimination, calibration, and reclassification. A total of 2,300 eligible cases were enrolled. Of them, 288 (12.5%) patients reached composite outcome and 214 (9.3%) patients reached ESRD during a median follow-up period of 30 months. Using the composite outcome for analysis, the Clinical, Limited, Full, and CKD models had relatively good performance with similar C statistics (0.81, 0.81, 0.82, and 0.82, respectively). While using ESRD as the end point, the four prediction models had better performance (all C statistics > 0.9). Furthermore, subgroup analysis showed that the models containing clinical and pathological variables (Full model and Limited model) had better discriminatory abilities than the models including only clinical indicators (Clinical model and CKD model) in low-risk patients characterized by higher baseline eGFR (≥60 ml/min/1.73 m^2^). In conclusion, we validated recently reported IgAN and CKD risk models in our Chinese IgAN cohort. Compared to pure clinical models, adding pathological variables will increase performance in predicting ESRD in low-risk IgAN patients with baseline eGFR ≥60 ml/min/1.73 m^2^.

## Introduction

Immunoglobulin A nephropathy (IgAN), first described by Berger in 1968, is the most common type of glomerulonephritis and an important cause of end-stage renal disease (ESRD) worldwide (1–3). Because of the heterogeneous prognostic nature of IgAN, it is important to identify high-risk patients at diagnosis not only for the selection for treatment strategies and clinical trials but also for patient health education (4–7).

In recent decades, dozens of clinical risk factors, including proteinuria, hypertension, and estimated glomerular filtration rate (eGFR), at the time of renal biopsy have been reported to be associated with worse renal prognosis in IgAN (8). In addition to clinical parameters at baseline, proteinuria or blood pressure during the first 2 years of follow-up after diagnosis also has clear correlations with the prognosis of IgAN (9). Among these risk factors, baseline eGFR was established as the most consistent indicator. An outstanding question in the field is whether combined pathological indicators, such as mesangial hypercellularity, endocapillary hypercellularity, segmental sclerosis, and interstitial fibrosis, can increase the accuracy of clinical indicators for prognosis prediction (9–11).

To date, several prediction models of IgAN progression have been established based on patients from different populations at different stages of renal function (9, 12–17). We previously established a clinical model (CLIN model) and a combined model containing both clinical and pathological variables (CLINPATH model), which had good performance in predicting the occurrence of ESRD at 10 years in the validation cohort (18). Later, a large-scale study of a combined multiethnic IgAN cohort performed by Barbour et al. established risk prediction models based on 3,927 IgAN patients (19). The clinical model in this study included proteinuria, blood pressure, and eGFR at renal biopsy. In addition to the clinical indicators, the limited model contained the MESTC histologic score, and the full model included age, medication, and racial/ethnic characteristics. The authors found that the limited model [area under the curve (AUC) = 0.80; 95% confidence interval (CI), 0.79–0.81] and full model (AUC = 0.82; 95% CI, 0.81–0.82) showed improved performance in predicting the composite outcome (defined as a 50% decline in eGFR or ESRD) compared to the clinical model (AUC = 0.78; 95% CI, 0.77–0.78). In addition, whether risk models of chronic kidney disease (CKD) can be used to predict the prognosis of IgAN is an interesting question. The study by Tangri developed and validated CKD risk models by including 8,391 Canadian CKD patients. Model 3 (C statistic, 0.91; 95% CI, 0.89–0.93), a clinical model, had good performance in predicting disease progression in patients with CKD stages 3 to 5 (20). Both studies by Barbour and Tangri studies have been further assessed and externally validated (21–23), but the prediction models would still benefit from additional external validation to improve confidence in using them in practice.

The objective of this study was to use our established multicenter Chinese IgAN cohort to conduct an independent external validation study of Barbour’s IgAN models and Tangri’s CKD model. We also compared the performance of pure clinical models (including clinical variables only) and combined models (including both clinical and pathological variables). We aimed to determine whether pathological parameters independently contribute to clinical models in predicting IgAN prognosis.

## Materials and Methods

### Ethics Approval and Consent to Participate

This study was performed in accordance with the Declaration of Helsinki and approved by the Ethics Research Committee of Ruijin Hospital, Medical School of Shanghai Jiaotong University. Written informed consent was collected from all participants prior to inclusion in the study.

### Participants

A multicenter collaborative cohort (six nephrology centers from teaching hospitals throughout the country) was established to represent Chinese patients with IgAN. All patients were recruited from six renal centers from 1985 to 2018. The recruitment criteria for the IgAN patients included the following: (1) IgAN was defined by a renal biopsy demonstrating dominant IgA deposition in the mesangium of glomeruli by immuno-fluorescence microscopy; (2) IgAN was not secondary to systemic diseases, such as Henoch-Schoünlein purpura, systemic lupus erythematosus, and liver disease; (3) the eGFR was ≥15 ml/min/1.73 m^2^ at diagnosis; (4) the minimum follow-up time was 6 months; (5) the age at biopsy was more than 18 years; and (6) an informed consent form was signed.

### Clinical and Pathologic Characteristics

All clinical and pathologic variables at the time of renal biopsy and during follow-up were collected. Age at biopsy, mean arterial blood pressure (MAP), serum creatinine (Scr), hemoglobin, eGFR (using the EPI equation), 24-h protein excretion, and renin–angiotensin system blocker (RASB) or glucocorticoid treatments were recorded. The severity of the renal damage was scored according to the Oxford MESTC classification (24). Three recently reported risk prediction models, including the clinical model, limited model, and full model with race/ethnicity (19), and one CKD risk prediction model (20), were used to calculate the risk of renal disease progression in individuals with IgAN.

### Outcomes and Definitions

The start of follow-up time was considered the date of renal biopsy. The primary renal outcome of our study was the combined outcome (the first occurrence of either a 50% decline in eGFR from that at biopsy or ESRD). The secondary outcome was defined as ESRD (eGFR < 15 ml/min/1.73 m^2^ or the need for dialysis/renal transplantation). Patients were censored at the time of meeting the endpoint criterion or loss to follow-up.

### Calculation of Predicted Risk and Risk Groups

To calculate the prediction risk of renal outcomes for each patient, the β coefficients from the original models of Barbour (19) and Tangri (20) were used **(** **Supplementary Table 1** **)**. Patients were categorized into four risk groups by the percentiles of linear predictors: low risk: <16th; intermediate risk: 16th to 50th; higher risk: 50th to 84th; and highest risk: > 84th percentile (19).

### Statistical Analysis

There are no reliable sample size recommendations for studies that validate prognostic models, but at least 100 events are recommended (25). Continuous data that are normally distributed or had a skewed distribution are expressed as the medians (interquartile range) or mean ± SD, respectively, and categorical data are expressed as the frequencies or percentages (%); probabilities of cumulative renal survival curves were generated by the Kaplan–Meier method. Prediction model performance was assessed using measures of model fit (Nagelkerke R2, Akaike information criterion (AIC), C statistic). Comparisons of the observed and predicted 5-year risk and 2-year risk of renal outcomes were analyzed separately. In addition, survival receiver operating characteristic (ROC) analysis was performed to evaluate the discriminatory ability of the scoring system after 5 years of follow-up. Reclassification improvement was quantified using the net reclassification improvement (NRI). Calibration refers to the agreement between observed outcomes and predictions, which was analyzed by the Hosmer–Lemeshow test in our study. Statistical analysis was performed using the ResourceSelection package (version 0.3-5), rms package (version 5.1-4), pROC package (version 1.16.1), and PredictABEL package (version 1.2-4) with the R statistical programming language (R, version 3.5.3; R Foundation for Statistical Computing, Vienna, Austria). Two-tailed *p*-values <.05 were considered statistically significant, except where otherwise indicated. The results are presented according to the TRIPOD guidelines for risk prediction models (**Supplementary Table 2**).

## Results

### Subject Characteristics

**Figure 1:**
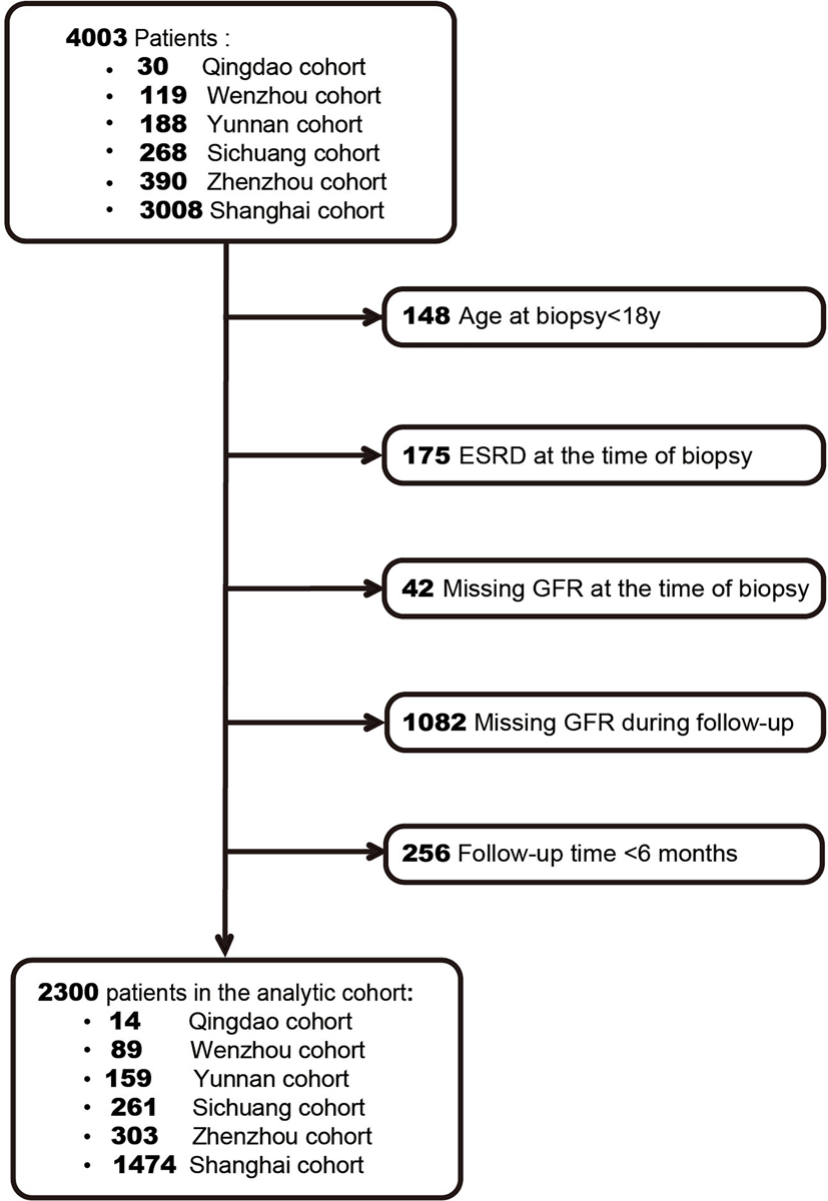
Flowchart of patient selection.

A total of 2,300 IgAN patients were finally enrolled based on the inclusion criteria (**Figure 1**). The characteristics of our cohort and the two original cohorts are summarized in **Table 1**. Our cohort included 1,106 males (48.1%), and the median age was 35 years (IQR, 28–44 years). The median values of baseline eGFR and 24-h proteinuria were 76.9 ml/min/1.73 m^2^ and 1.3 g/day, respectively. Among the included patients, 73.7% received RASB treatment and 59.8% received glucocorticoid treatment after diagnosis. During the median follow-up time of 2.5 years, 288 patients (12.5%) had a renal composite outcome, and 214 patients progressed to ESRD (9.3%).

**Table 1:** Characteristics of patients in our external validation cohort, Barbour’s derivation cohort, and Tangri’s derivation cohort.^a^

Characteristic	External validation cohort	Barbour’s derivation cohort	Tangri’s derivation cohort
Patients, N	2,300	2,781	3,449
Follow up, median, years	2.5	4.8	2.1
Death	10 (0.4)	35 (1.2)	N/A
Age, median (IQR), or mean ± SD, years	35 (28–44)	35.6 (28.2–45.4)	70 ± 14
Male sex	1,106 (48.1)	1,608 (57.8)	1,946 (56)
Race/ethnicity			
Chinese	2,300 (100)	1,021 (36.7)	0 (0)
Creatinine level at biopsy, median (IQR), μmol/L	95 (71–134)	92.0 (70.7–123.8)	N/A
eGFR at biopsy, median (IQR), or mean ± SD, mL/min/1.73 m^2^	76.9 (50.1–103.6)	83.0 (56.7–108.0)	36 ± 13
<15	0 (0)	0 (0)	220 (6)
15–30	155 (6.7)	142 (5.1)	926 (27)
30–60	632 (27.5)	657 (23.6)	2,303 (67)
60–90	648 (28.2)	800 (28.8)	0 (0)
>90	865 (37.6)	1182 (42.5)	0 (0)
MAP at biopsy, median (IQR), mmHg	96.3 (88.3–105.7)	96.7 (88.7–106.3)	N/A
Hemoglobin, median (IQR), or mean ± SD, g/dL	12.9 (11.6–14.3)	N/A	12.4 ± 1.8
^b^Proteinuria at biopsy, Median (IQR), g/d	1.3 (0.7–2.6)	1.2 (0.7–2.2)	N/A
<0.5	345 (15.2)	383 (13.9)	N/A
0.5–1	511 (22.5)	772 (28.1)	N/A
1–2	625 (27.5)	817 (29.7)	N/A
2–3	333 (14.6)	360 (13.1)	N/A
>3	460 (20.2)	415 (15.1)	N/A
^c^MESTC histologic score			
M1	779 (40.9)	1,054 (38.0)	N/A
E1	623 (32.7)	478 (17.3)	N/A
S1	1,385 (72.7)	2,137 (77.0)	N/A
T1	457 (24.0)	686 (24.7)	N/A
T2	272 (14.3)	128 (4.6)	N/A
C1	757 (39.8)	N/A	N/A
C2	122 (6.4)	N/A	N/A
^d^RASB use after biopsy (during follow-up)	1,593 (73.7)	2,400 (86.7)	N/A
^d^Glucocorticoid use after biopsy	1,292 (59.8)	1,209 (43.5)	N/A
Primary outcome			
50% decline in eGFR	264 (11.5)	420 (15.1)	N/A
ESRD (kidney failure)	214 (9.3)	372 (13.4)	386 (11)
Total primary outcome	288 (12.5)	492 (17.7)	N/A

IQR, interquartile range; eGFR, estimated glomerular filtration rate calculated by CKD-EPI formula; MAP, mean arterial blood pressure; MESTC, mesangial (M) and endocapillary (E) hypercellularity, segmental sclerosis (S), interstitial fibrosis and tubular atrophy (T) and crescents (C); RASB, renin–angiotensin system blocker; ESRD, end-stage renal disease; N/A, not available.

a Unless otherwise indicated, data are reported as number (percentage) of patients.

b A total of 2,274 has proteinuria records.

c A total of 1,904 has OXFORD-MESTC score.

d A total of 2,161 has treatment records.

### Performance of the IgAN Prediction Tool in Two Renal Outcomes

The goodness of fit and statistics for discrimination for all models at 5 years after biopsy are shown in **Tables 2** and **3**, respectively. Using the composite outcome as an endpoint, the clinical model, including eGFR, proteinuria, and MAP, performed well (C statistic, 0.81; R^2^, 0.23). The C statistic and R^2^ were not significantly improved after adding pathological indicators in the limited model (C statistic, 0.82; R^2^, 0.27) or medication and other predictors in the full model (C statistic, 0.82; R^2^, 0.27). The AIC was also similar among the clinical, limited, and full models (712.10 and 689.33 *vs*. 687.68, respectively). Moreover, continuous net reclassification improvement [cNRI; full model *vs*. clinical model: 0.36 (95% CI, 0.18–0.55); full model *vs*. limited model: 0.08 (95% CI, -0.11–0.27)] indicated the significantly improved classification performance of the models that included clinical and pathological indicators compared with the models that only included clinical indicators at 5 years of follow-up (**Table 3**). **Supplementary Figure 1** shows the mean predicted risk probability of the composite outcome against the observed risk over the follow-up period. The full model with race was calibrated well, with a mild underestimation in the low-, intermediate-, and highest-risk groups and mild overestimation in the higher-risk group.

**Table 2:** The goodness of fit for different models predicting the composite outcome (50% GFR declined or ESRD) and ESRD at 5 years.

	Clinical models		Clinical and pathology models	
Outcomes	Clinical Model^a^	CKD model^b^	Limited Model^a^	Full Model^a^
**ESRD or 50% GFR decreased at 5 years**				
AIC	712.1	706.28	689.33	687.68
R2	0.23	0.24	0.27	0.27
C statistic	0.81 (0.76–0.86)	0.81 (0.76–0.86)	0.82 (0.77–0.87)	0.82 (0.78–0.87)
**ESRD at 5 years**				
AIC	453.14	450.11	441.26	440.24
R2	0.31	0.31	0.32	0.31
C statistic	0.90 (0.86–0.93)	0.90 (0.86–0.94)	0.91 (0.88–0.94)	0.91 (0.88–0.95)

eGFR, estimated glomerular filtration rate; MAP, mean arterial blood pressure; MESTC, mesangial (M) and endocapillary hypercellularity (E), segmental sclerosis (S), interstitial fibrosis and tubular atrophy (T) and crescents(C); RASB, renin-angiotensin system blocker; AIC, Akaike information criterion; ref, reference.

A total of 1,764 patients have comprehensive treatment and clinical, histological records. Higher values for C statistic and R^2^ and lower values for AIC indicate better models.

a Clinical Model, Limited Model and Full Model from Barbour’s study contain clinical variables only or clinical, pathological, medication use, and ethnic variables, respectively (19).

b CKD Model from Tangri’s study contains clinical variables including baseline GFR, age, gender, and proteinuria (CKD model 3) (20).

**Table 3:** For total group, comparison of models’ discrimination performance in the validation cohort for predicting the risk for two outcomes (ESRD or 50% GFR decreased; ESRD) at 5 years after biopsy.

	Clinical Model	CKD Model	Limited Model
**5 years at 50% GFR decrease or ESRD**			
**cNRI**			
CKD model	0.10 (-0.08–0.29)	–	
Limited model	0.29 (0.10–0.47)^b^	0.40 (0.22–0.58)^a^	–
Full model	0.36 (0.18–0.55)^a^	0.40 (0.21–0.58)^a^	0.08 (-0.11–0.27)
**5 years at ESRD**			
**cNRI**			
CKD model	0.004 (-0.22–0.23)	–	
Limited model	0.41 (0.19–0.63)^a^	0.42 (0.20–0.63)^a^	–
Full model	0.36 (0.15–0.58)^a^	0.41 (0.19–0.62)^a^	-0.02 (-0.25–0.21)

cNRI, continuous net reclassification improvement; 95% CI, 95% confidence interval.

a p < 0.001.

b p < 0.05.

**Figure 2:**
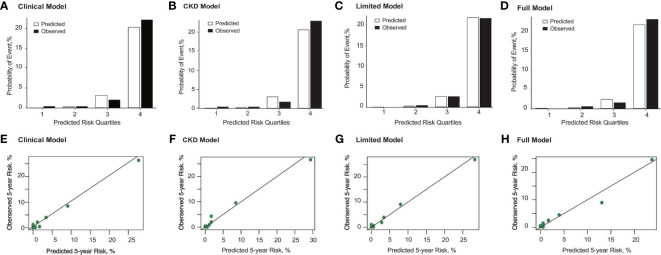
Observed *vs*. predicted probability and calibration curve of ESRD at 5 years using the Clinical Model **(A, E)**, CKD Model **(B, F)**, Limited Model **(C, G)**, and Full Model **(D, H)**. The predicted and observed event probability estimates represent the mean predicted probability from risk-prediction model and the mean observed probability from the population divided into quartiles of predicted probability. For those models, risk groups were based on the 16th (lowest risk), 16th to 50th (intermediate risk), 50th to 84th (higher risk), and higher than 84th (highest risk) percentiles of the linear predictor. The mean predicted probability (%) *vs*. observed probability (%) categories for quartiles 1 through 4 correspond with 0.02% *vs*. 0.35%, 0.25% *vs*. 0.33%, 3.14% *vs*. 2.00%, and 20.36% *vs*. 22.26%, respectively, for the Clinical Model; 0.03% *vs*. 0.35%, 0.24% *vs*. 0.33%, 3.01% *vs*. 1.67%, and 20.65% *vs*. 22.97%, respectively, for the CKD Model; 0.03% *vs*. 0.00%, 0.21% *vs*. 0.33%, 2.53% *vs*. 2.50%, and 21.74% *vs*. 21.55%, respectively, for the Limited Model; and 0.03% *vs*. 0.00%, 0.22% *vs*. 0.50%, 2.42% *vs*. 1.50%, and 21.95% *vs*. 23.32%, respectively, for the Full Model. In addition, the Hosmer–Lemeshow test presented that the full model with race had p value > 0.05, which means the goodness of model fit is acceptable.

Indeed, the three IgAN models used for predicting ESRD performed better (all C statistics > 0.9) than that used to predict the composite outcome (**Table 2**). Compared with the clinical model, the full model with race also demonstrated significant improvement in risk reclassification for predicting 5-year risk, with an NRI of 0.36 (95% CI, 0.15 to 0.58, **Table 3**). Overall, the three models mildly underestimated the risk within 5 years in the highest-risk group (**Figure 2**). In addition, we validated the performance of those models in predicting the 2-year renal outcome and found that they could also effectively predict short-term prognosis (**Supplementary Tables 3** and **4**).

### Performance of the CKD Prediction Tool in Two Renal Outcomes

**Figure 3:**
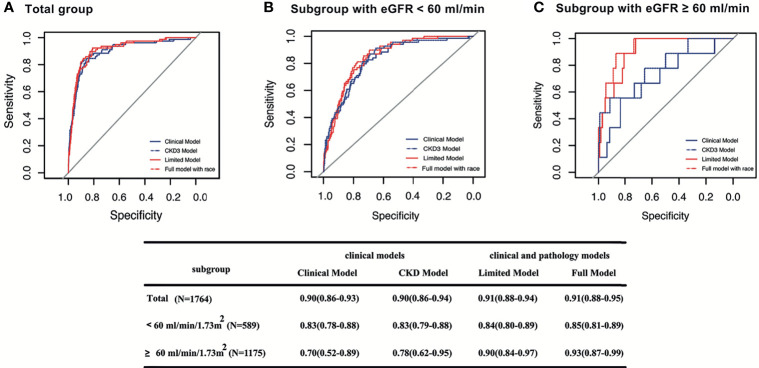
Survival ROC curves of four models for predicting ESRD at 5 years in different subgroups. The area the under the curve (AUC) and 95% CI of the Clinical Model, CKD Model, Limited Model, and Full Model, respectively, in total patients **(A)**; in patients with baseline eGFR < 60 ml/min/1.73 m^2^ **(B)**; in patients with baseline eGFR ≥ 60 ml/min/1.73 m^2^ **(C)**.

We next evaluated the performance of the CKD risk prediction model predicting different renal outcomes in IgAN patients given the CKD-like nature of IgAN. As a model containing only clinical indicators, CKD model 3 also had excellent performance in predicting ESRD (C statistic, 0.90; 95% CI, 0.86–0.94) and relatively good performance in predicting composite outcomes (C statistic, 0.81; 95% CI, 0.76–0.86) in our IgAN patients (**Table 2**). Using ESRD as the renal outcome, the R^2^ (0.31) and AIC (450.11) were also similar to those of the above clinical models (**Table 2**). The clinical models based on baseline eGFR and various other clinical parameters exhibited good performance. In addition, the difference between the observed and predictive probabilities (**Figure 2**) and the ROC curve (**Figure 3**) for predicting ESRD at 5 years in the CKD model were similar to those of the above IgAN models.

The IgAN models and the CKD model performed better in predicting ESRD than in predicting the composite endpoint. Considering that ESRD is a robust renal outcome, it was used for further analysis.

### Subgroup Analysis of the Four Models for Predicting ESRD

A subgroup analysis of the entire cohort was used to evaluate the performance of the four models in patients from different subgroups (**Supplementary Figure 2**). Either clinical models or clinical/pathological models had similar discriminatory abilities within subgroups defined by age, sex, proteinuria, or glucocorticoid treatment. Compared with that of the clinical models, the improved performance of the combined models after adding pathological indicators was limited, even for patients at high risk characterized by lower baseline eGFR (<60 ml/min/1.73 m^2^).

### Comparison of Models for Predicting ESRD in Patients With Lower Risk

For patients at low risk characterized by baseline eGFR ≥60 ml/min/1.73 m^2^, we compared the performance of all models based on ROC analysis. The full model provided considerably improved discriminative power compared with the other three previously proposed models in predicting ESRD at 5 years of follow-up [C statistic of 0.93 (95% CI, 0.87–0.99) for the full model, 0.90 (95% CI, 0.78–0.97) for the limited model, 0.70 (95% CI, 0.52–0.89) for the clinical model, 0.78 (95% CI, 0.62–0.95) for the CKD model]. Pathological variables that added predictive value to the clinical variables were observed only in low-risk IgAN patients characterized by a baseline eGFR ≥ 60 ml/min/1.73 m^2^ (**Figure 3**).

## Discussion

Clinical challenges of IgAN include accurately stratifying patients, helping clinicians to identify high-risk patients to enhance treatment, and avoiding unnecessary hormone and immunosuppressive therapies for low-risk patients. Recently, with the efforts of clinical nephrologists, multiple risk models have been established. Some models include baseline or follow-up clinical parameters, such as eGFR, proteinuria, and blood pressure, and some models add pathological parameters to the clinical models to establish combined models. These predictive models still benefit from other external validations, thereby increasing the confidence in their clinical use. In addition, whether pathological parameters, such as Oxford MEST predictors, can enhance the predictive value of clinical parameters for the prognosis of patients with IgA nephropathy remains controversial.

In this study, we assessed the performance of international IgAN prediction tools and another CKD model by external validation in a large, multicenter Chinese IgAN cohort. Relative to those in the derivation cohort of the international IgAN prediction tools, our follow-up time was shorter, and the incidence of a 50% decline in eGFR or development of ESRD was lower (12.5% *versus* 17.7%). Compared to the derivation cohort of the CKD model, our cohort had a lower proportion of patients with baseline eGFR < 30 ml/min/1.73 m^2^, and fewer patients progressed to kidney failure/ESRD (9.3% *versus* 11%) during the follow-up period.

Indeed, we still found that the four models performed well at predicting the 5-year risk of renal outcomes. Compared with composite outcomes, better performance of those tools used for predicting ESRD at 5 years was observed. These models also performed relatively well at predicting short-term prognosis. In addition, we found that the clinical models based on baseline eGFR and various other clinical parameters had good performance, as did the combined model that included clinical and pathological indicators. For IgAN patients at low risk characterized by higher baseline eGFR, adding pathological variables could enhance the discriminatory ability of models that contain only clinical variables. Finally, after application to patients at low risk, the full model had the best performance in predicting ESRD among the four reported models.

Among patients with IgAN, there can be considerable heterogeneity in the risk for progression to kidney failure. Risk factors associated with IgAN progression have gained increasing attention over the last two decades (26–29). The emerging literature suggests improved patient outcomes with individualized risk prediction models (30–34). The availability of these risk prediction tools has led to better adherence to treatment guidelines and encouraged individual decision making (32–34). Despite these benefits, the lack of easily applicable and externally validated models has delayed the widespread integration of risk prediction in all fields of medicine (35, 36). We confirmed that the models rely on clinical data and histological markers of IgAN severity to predict the early risk of kidney failure at 5 years. Similar to Barbour (37, 38), we also confirmed that a lower estimated eGFR, more severe proteinuria, and male gender predict faster progression to kidney failure. In addition, a higher percentage of tubular injury and segmental sclerosis also predict a higher risk of kidney failure. These markers may enable a better estimate of the underlying processes of disease (39, 40).

Considering that those laboratory and pathological markers have been associated with the progression of IgAN, risk prediction models integrate them in different combinations. Based on our data, the performances of all models according to ROC analysis were compared. The clinical models and combined models showed similar performance in predicting ESRD after 5 years of follow-up. However, for patients at low risk characterized by higher baseline eGFR (≥60 ml/min/1.73 m^2^), the pathological variables could add predictive value to clinical variables, likely because the contribution of pathological indicators to ESRD prediction is diminished by the subsequent use of immunosuppressive therapy in high-risk patients characterized by lower eGFR.

Risk prediction models have important implications for clinical practice, research, and public health policy. Different risk thresholds could be used to triage patients for decision-making. For example, primary care physicians could manage lower-risk patients without additional testing or treatment of complications, whereas higher-risk patients could receive more intensive testing, intervention, and early nephrology care (41). Furthermore, the risk prediction model could be used to select higher-risk patients for enrollment into clinical trials and for the evaluation of risk-treatment interactions. In addition, the risk prediction model may be useful for identifying high-risk patients for public health interventions, thereby improving the cost-effectiveness of medical care.

The strength of our study is that we added a strict primary endpoint of ESRD, which is more relevant than other common endpoints based on declined eGFR or CKD stage. Specifically, for patients at low risk characterized by higher baseline eGFR (≥60 ml/min/1.73 m^2^), using the full model to predict ESRD at 5 years should be more precise. Additionally, both the CKD model and IgAN model exhibited similar performance in our IgAN cohort. We still need to improve the predictive ability of the models by adding IgAN-specific biomarkers, such as HAA-IgA1 levels. Alternatively, considering the genetic background of IgAN (1, 42–45), adding genetic risk factors (29, 46) involved in disease progression could also be useful for improving risk prediction of disease progression.

Our analysis also has limitations. We did not explicitly model the risk of all-cause mortality in our IgAN population because the number of deaths in our cohort might have been underestimated. In addition, disease duration and treatment information prior to renal biopsy were incomplete, thus we did not involve these data for the analysis. Moreover, although the CKD model from the study by Tangri was evaluated and performed well in our Chinese IgAN cohort, more CKD cohorts are still needed to validate it. Additionally, as this study was a multicenter cohort study, the heterogeneity of the study population is a limitation. The lack of detailed data on systematic therapies from all the centers is another limitation.

## Conclusion

In summary, we validated recently reported highly accurate predictive models for the progression of IgAN to kidney failure. Especially for patients at low risk characterized by higher baseline eGFR (≥60 ml/min/1.73 m^2^), an improvement in model performance was observed after adding histological indicators to these clinical indicators.

## Data Availability Statement

The original contributions presented in the study are included in the article/**Supplementary Material**. Further inquiries can be directed to the corresponding author.

## Ethics Statement

The studies involving human participants were reviewed and approved by the Ethics Research Committee from Ruijin Hospital, Medical School of Shanghai Jiaotong University. The patients/participants provided their written informed consent to participate in this study.

## Author Contributions

JXi designed and was responsible for the study. YO analyzed the data and drafted the paper. ZZ, GL, HL, FX, LS, ZC, SY, YJ, JXu, MS, HH, WD, ZF, XP, WM, and NC collected the data. JXi revised the paper. All authors approved the final version of the manuscript. All authors contributed to the article and approved the submitted version.

## Funding

This work was supported by the Major International (Regional) Joint Research Program of National Natural Science Foundation of China (No:8211001014), the National Natural Science Foundation of China (Nos. 81870460, 81570598, 81370015, 81900656), “Excellent Academic Leader” by Shanghai Science and Technology Commission (No: 21XD1402000). Shanghai Jiao Tong University “Jiaotong Star” Plan Medical Engineering Cross Research Key Project (No: YG2019ZDA18, YG2019QNA37), Shanghai Shenkang Hospital Development Center “Three-year Action Plan for Promoting Clinical Skills and Clinical Innovation in Municipal Hospitals” (No : SHDC2020CR6017), Science and Technology Innovation Action Plan of Shanghai Science and Technology Committee (No. 17441902200), Shanghai Municipal Education Commission, Gaofeng Clinical Medicine Grant (No. 20152207), Shanghai Jiao Tong University School of Medicine, Multi-Center Clinical Research Project (No: DLY201510), Shanghai Health and Family Planning Committee Hundred Talents Program (No: 2018BR37), and Shanghai Municipal Key Clinical Specialty (shslczdzk02502). No funding bodies had any role in study design, data collection and analysis, decision to publish, or preparation of the manuscript.

## Conflict of Interest

The authors declare that the research was conducted in the absence of any commercial or financial relationships that could be construed as a potential conflict of interest.

## Publisher’s Note

All claims expressed in this article are solely those of the authors and do not necessarily represent those of their affiliated organizations, or those of the publisher, the editors and the reviewers. Any product that may be evaluated in this article, or claim that may be made by its manufacturer, is not guaranteed or endorsed by the publisher.
