# Supplementary material for: A Validation Study Comparing Risk Prediction Models of IgA Nephropathy
Source: Front Immunol. 2021 Oct 15;12:753901. doi: 10.3389/fimmu.2021.753901 (PMC8554097; doi:10.3389/fimmu.2021.753901)
Supplement: Supplementary file 1 [file DataSheet_1.docx]

**Supplementary Material**

| Supplementary Table1. Characteristics and β value of the four studied prognostic models. | | | | |
| --- | --- | --- | --- | --- |
| **Characteristic** | **Clinical Model ^(1)^** | **Limited Model ^(1)^** | **Full model with race ^(1)^** | **CKD Model ^(2)*^** |
|  |  |  |  |  |
| Baseline eGFR | -0.35 | -0.26 | -0.35 | -0.56 |
| Age |  |  | -0.02 | -0.22 |
| Male sex |  |  |  | 0.23 |
| Proteinuria | 0.52 | 0.48 | -0.09 |  |
| Spot urine ACR |  |  |  | 0.47 |
| MAP | 0.008 | 0.005 | -0.0002 |  |
| Histologic score |  |  |  |  |
| M1 |  | 0.29 | 0.16 |  |
| E1 |  | -0.12 | -0.13 |  |
| S1 |  | 0.14 | 0.1 |  |
| T1 |  | 0.82 | 0.61 |  |
| T2 |  | 1.11 | 1.19 |  |
| Proteinuria x T1 |  |  | 0.11 |  |
| Proteinuria x T2 |  |  | -0.34 |  |
| Proteinuria x MAP |  |  | 0.01 |  |
| Race: |  |  |  |  |
| Chinese (<=3 years) |  |  | -0.4 |  |
| Chinese (>3 years) |  |  | 0.82 |  |
| Japanese |  |  |  |  |
| Caucasian |  |  |  |  |
| RASB |  |  | 0.25 |  |
| Immunosuppression |  |  | -0.23 |  |

Note: Since all our patients are Chinese, the coefficients for Japanese and Caucasians in the formula are not included in the calculation.

*The article of CKD model provides Hazard Ratio; we transfer it to beta by R software [log (HR) = β].

Abbreviation: eGFR, estimated glomerular filtration rate; ACR, albumin-to-creatinine ratio; MAP, mean arterial blood pressure; MESTC, mesangial (M) and endocapillary (E) hypercellularity, segmental sclerosis (S), interstitial fibrosis and tubular atrophy (T) and crescents(C); RASB, renin-angiotensin system blocker.

**References:**

(1). Barbour SJ, Coppo R, Zhang H, Liu ZH, Suzuki Y, Matsuzaki K, et al: Evaluating a New International Risk-Prediction Tool in IgA Nephropathy. JAMA Intern Med. 179(7):942-952,2019;

(2). Tangri N, Stevens LA, Griffith J, Tighiouart H, Djurdjev O, Naimark D, et al: A predictive model for progression of chronic kidney disease to kidney failure. JAMA. 305(15):1553-1559,2011;

| **Supplementary Table S2. TRIPOD checklist for prediction model validation (V).**   \| **Section/Topic** \| **Item** \| **Checklist Item** \| **Page** \| \| --- \| --- \| --- \| --- \| \| **Title and abstract** \| \| \| \| \| Title \| 1 \| Identify the study as developing and/or validating a multivariable prediction model, the target population, and the outcome to be predicted. \| P1 \| \| Abstract \| 2 \| Provide a summary of objectives, study design, setting, participants, sample size, predictors, outcome, statistical analysis, results, and conclusions. \| P2 \| \| **Introduction** \| \| \| \| \| Background and objectives \| 3a \| Explain the medical context (including whether diagnostic or prognostic) and rationale for developing or validating the multivariable prediction model, including references to existing models. \| P2-3 \| \| 3b \| Specify the objectives, including whether the study describes the development or validation of the model or both. \| P4 \| \| **Methods** \| \| \| \| \| Source of data \| 4a \| Describe the study design or source of data (e.g., randomized trial, cohort, or registry data), separately for the development and validation data sets, if applicable. \| P4 \| \| 4b \| Specify the key study dates, including start of accrual; end of accrual; and, if applicable, end of follow-up. \| P4 \| \| Participants \| 5a \| Specify key elements of the study setting (e.g., primary care, secondary care, general population) including number and location of centres. \| P4 \| \| 5b \| Describe eligibility criteria for participants. \| P4 \| \| 5c \| Give details of treatments received, if relevant. \| P4 \| \| Outcome \| 6a \| Clearly define the outcome that is predicted by the prediction model, including how and when assessed. \| P4-5 \| \| 6b \| Report any actions to blind assessment of the outcome to be predicted. \| n/a \| \| Predictors \| 7a \| Clearly define all predictors used in developing or validating the multivariable prediction model, including how and when they were measured. \| P4 \| \| 7b \| Report any actions to blind assessment of predictors for the outcome and other predictors. \| n/a \| \| Sample size \| 8 \| Explain how the study size was arrived at. \| P5 \| \| Missing data \| 9 \| Describe how missing data were handled (e.g., complete-case analysis, single imputation, multiple imputation) with details of any imputation method. \| P5 \| \| Statistical analysis methods \| 10c \| For validation, describe how the predictions were calculated. \| P5 \| \| 10d \| Specify all measures used to assess model performance and, if relevant, to compare multiple models. \| P5 \| \| 10e \| Describe any model updating (e.g., recalibration) arising from the validation, if done. \| n/a \| \| Risk groups \| 11 \| Provide details on how risk groups were created, if done. \| P5 \| \| Development vs. validation \| 12 \| For validation, identify any differences from the development data in setting, eligibility criteria, outcome, and predictors. \| P4-5 \| \| **Results** \| \| \| \| \| Participants \| 13a \| Describe the flow of participants through the study, including the number of participants with and without the outcome and, if applicable, a summary of the follow-up time. A diagram may be helpful. \| P6 \| \| 13b \| Describe the characteristics of the participants (basic demographics, clinical features, available predictors), including the number of participants with missing data for predictors and outcome. \| P6 \| \| 13c \| For validation, show a comparison with the development data of the distribution of important variables (demographics, predictors and outcome). \| P8 \| \| Model performance \| 16 \| Report performance measures (with CIs) for the prediction model. \| P6-7 \| \| Model-updating \| 17 \| If done, report the results from any model updating (i.e., model specification, model performance). \| n/a \| \| **Discussion** \| \| \| \| \| Limitations \| 18 \| Discuss any limitations of the study (such as nonrepresentative sample, few events per predictor, missing data). \| P10 \| \| Interpretation \| 19a \| For validation, discuss the results with reference to performance in the development data, and any other validation data. \| P8-9 \| \| 19b \| Give an overall interpretation of the results, considering objectives, limitations, results from similar studies, and other relevant evidence. \| P9-10 \| \| Implications \| 20 \| Discuss the potential clinical use of the model and implications for future research. \| P10-11 \| \| **Other information** \| \| \| \| \| Supplementary information \| 21 \| Provide information about the availability of supplementary resources, such as study protocol, Web calculator, and data sets. \| P22 \| \| Funding \| 22 \| Give the source of funding and the role of the funders for the present study. \| P11 \|   n/a, not available  **Supplementary Table S3. The goodness fit for different models predicting ESRD at 2 years. ^a^** | | | | | |
| --- | --- | --- | --- | --- | --- | --- | --- | --- | --- | --- | --- | --- | --- | --- | --- | --- | --- | --- | --- | --- | --- | --- | --- | --- | --- | --- | --- | --- | --- | --- | --- | --- | --- | --- | --- | --- | --- | --- | --- | --- | --- | --- | --- | --- | --- | --- | --- | --- | --- | --- | --- | --- | --- | --- | --- | --- | --- | --- | --- | --- | --- | --- | --- | --- | --- | --- | --- | --- | --- | --- | --- | --- | --- | --- | --- | --- | --- | --- | --- | --- | --- | --- | --- | --- | --- | --- | --- | --- | --- | --- | --- | --- | --- | --- | --- | --- | --- | --- | --- | --- | --- | --- | --- | --- | --- | --- | --- | --- | --- | --- | --- | --- | --- | --- | --- | --- | --- | --- | --- | --- | --- | --- | --- | --- | --- | --- | --- | --- | --- | --- | --- | --- | --- | --- | --- | --- | --- | --- | --- | --- | --- | --- | --- | --- | --- | --- |
|  | **clinical models** | |  | **clinical and pathology models** | |
| **Outcomes** | **Clinical Model ^b^** | **CKD Model ^c^** |  | **Limited Model ^b^** | **Full Model ^b^** |
| **ESRD or 50%GFR decreased at 2 years** | |  |  |  |  |
| AIC | 393.61 | 393.12 |  | 383.39 | 384.75 |
| R2 | 0.20 | 0.20 |  | 0.25 | 0.25 |
| C statistic | 0.83(0.76-0.90) | 0.83(0.75-0.91) |  | 0.84(0.77-0.91) | 0.84(0.77-0.91) |
| **ESRD at 2 years** |  |  |  |  |  |
| AIC | 206.59 | 208.11 |  | 202.98 | 205.14 |
| R2 | 0.28 | 0.27 |  | 0.34 | 0.33 |
| C statistic | 0.94(0.89-0.99) | 0.94(0.89-0.99) |  | 0.95(0.90-0.99) | 0.95(0.91-0.99) |
| eGFR, estimated glomerular filtration rate; MAP, mean arterial blood pressure; MESTC, mesangial (M) and endocapillary. | | | | | |
| hypercellularity(E), segmental sclerosis (S), interstitial fibrosis and tubular atrophy (T) and crescents(C); RASB, renin-angiotensin system blocker. | | | | | |
| AIC, Akaike Information Criterion; ref, reference. | | | | | |
| ^a^Unless otherwise indicated, data are reported as Hazard ratio (95% confidence interval); 1764 patients has comprehensive treatment and clinical, histological records. | | | | | |
| ^b^Clinical Model, Limited Model and Full Model with Race from Barbour's study contain clinical variables only or clinical, pathological, medication use and ethnic variables, respectively [JAMA Intern Med (2019) 179:942–52. doi: 10.1001/jamainternmed.2019.0600] | | | | | |
| ^c^CKD Model from Tangri's study contains clinical variables including baseline GFR, age, gender and proteinuria (CKD model 3) [JAMA (2011) 305:1553–9.doi: 10.1001/jama.2011.451] ; | | | | | |
| Higher values for C statistic and R^2^, and lower values for AIC indicate better models. | |  |  |  |  |

| **Supplementary Table S4. Comparison of Models' discrimination performance in the Validation Cohort for predicting the risk for two outcomes (ESRD; ESRD/50%GFR decreased) at 2 years after biopsy**. | | | | |
| --- | --- | --- | --- | --- |
|  | **Clinical Model** | **CKD Model** | **Limited Model** |  |
| **50%GFR decrease or ESRD at 2 years** | |  |  |  |
| **cNRI** |  |  |  |  |
| CKD Model | 0.04[-0.23 - 0.31] | - |  |  |
| Limited Model | 0.34[0.07 - 0.61] ^b^ | 0.56 [0.31- 0.81] ^a^ | - |  |
| Full Model | 0.42[0.15 - 0.68] ^b^ | 0.55[0.30 - 0.80] ^a^ | 0.07[-0.20 - 0.34] |  |
| **ESRD at 2 years** |  |  |  |  |
| **cNRI** |  |  |  |  |
| CKD Model | -0.22[-0.57- 0.13] | - |  |  |
| Limited Model | 0.46 [0.12 - 0.80] ^b^ | 0.51[0.17 - 0.84] ^b^ | - |  |
| Full Model | 0.39 [0.05 - 0.73] ^b^ | 0.41[0.07- 0.75] ^b^ | -0.14 [-0.49 - 0.21] |  |
| cNRI, Continuous net reclassification improvement ; 95%CI, 95% confidence interval; | | | | |
| ^a^P<0.001  ^b^P<0.05 |  |  |  |  |
|  |  |  |  |  |

**
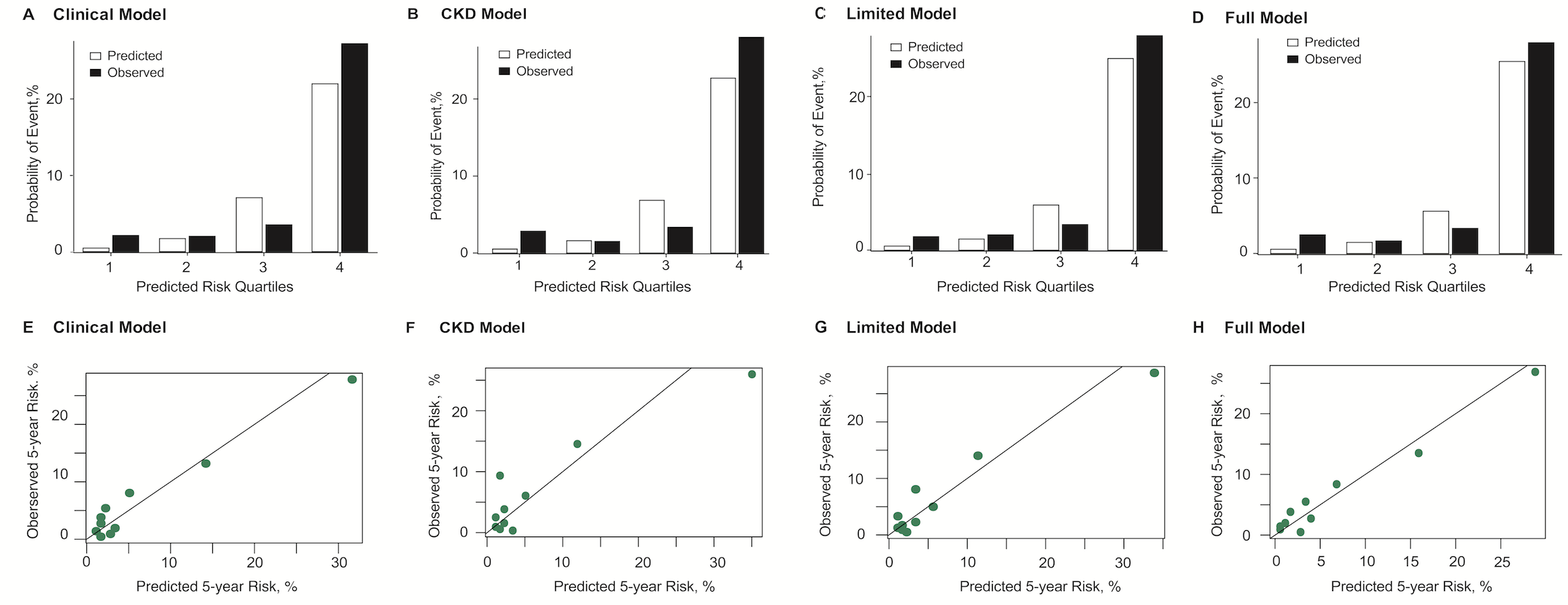
**

**Supplementary Figure S1.Observed vs. Predicted probability and Calibration plot of composite outcome at 5 years using Clinical Model (A and E), CKD Model (B and F), Limited Model(C and G) and Full Model (D and H).** The predicted and observed event probability estimates represent the mean predicted probability from risk-prediction model and the mean observed probability from the population divided into quartiles of predicted probability. For those models, predicted risk categories for quartiles 1 through 4 correspond with less than 16th (lowest risk), 16th to 50th (intermediate risk), 50th to 84th (higher risk) and higher than 84th (highest risk) percentiles.

**
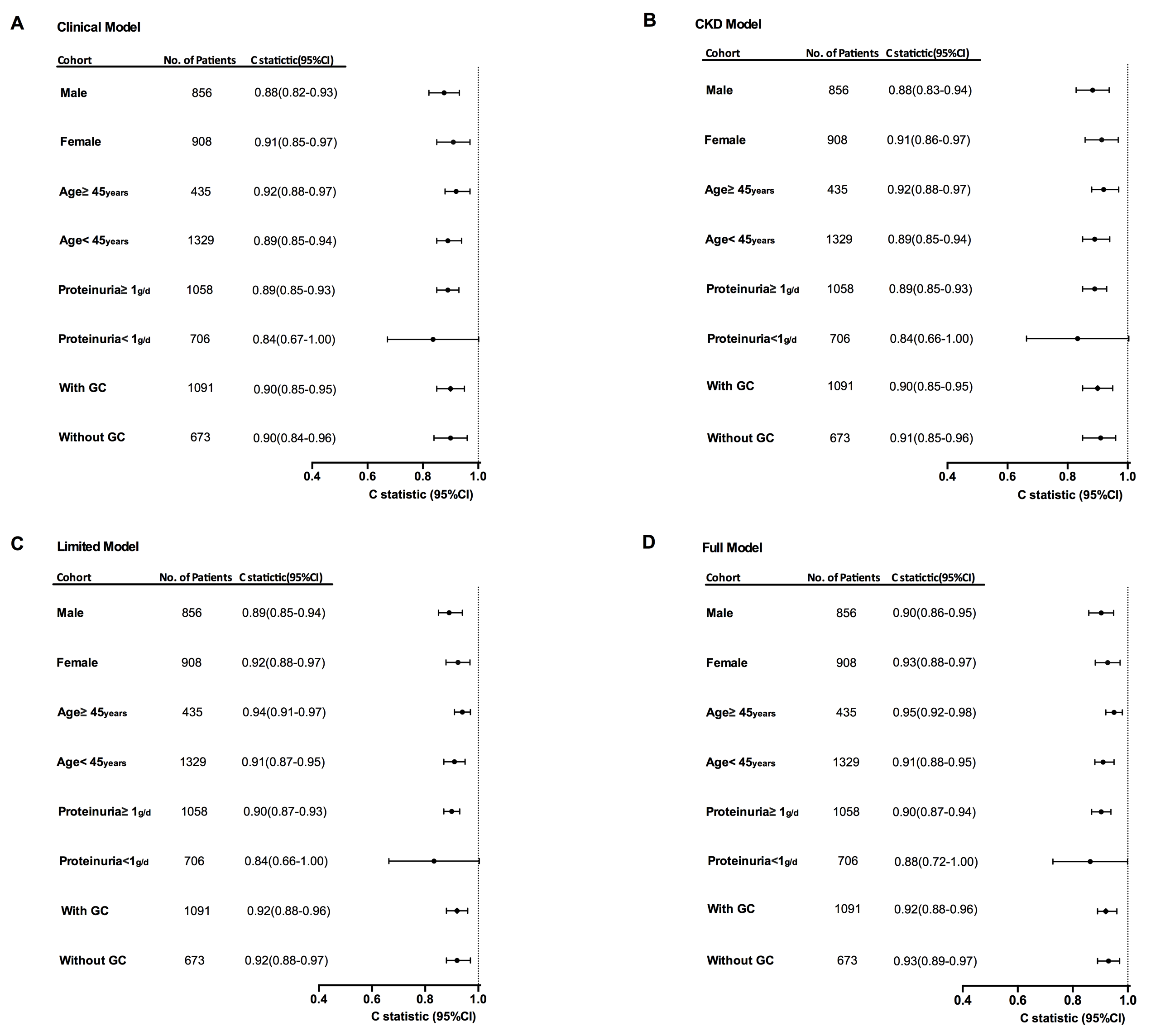
**

**Supplementary Figure S2. Discrimination statistics (C statistics) for predicting probability of ESRD at 5 years by Clinical Model (A), CKD Model(B), Limited Model(C) and Full Model (D) in different subgroups. 95%CI, 95% confidence interval. GC, glucocorticoid.**
